# Health and social needs of asylum seekers and Ukrainian refugees in Lithuania: A mixed-method protocol

**DOI:** 10.3389/fpubh.2022.1025446

**Published:** 2023-01-10

**Authors:** Rabie Adel El Arab, Rita Urbanavice, Agne Jakavonyte-Akstiniene, Marija Skvarcevskaja, Donatas Austys, Jose Tomas Mateos, Erica Briones-Vozmediano, Esther Rubinat-Arnaldo, Natalja Istomina

**Affiliations:** ^1^Faculty of Nursing and Physiotherapy, University of Lleida, Lleida, Spain; ^2^Institute for Biomedical Research (IRBLleida), Healthcare Research Group (GRECS), Lleida, Spain; ^3^Health and Social Services for Asylum Seekers Research Group, Vilnius University, Vilnius, Lithuania; ^4^Institute of Health Sciences, Faculty of Medicine, Vilnius University, Vilnius, Lithuania

**Keywords:** healthcare services, social needs, Lithuania, asylum seekers, refugees

## Abstract

Refugees, asylum seekers, and migrants often do not end up in the places they expected. Because of the pandemic impacts, their exposure to COVID-19 may be increased as a result of crowded camps and detention centers. A total of 4,537 undocumented migrants entered Lithuania *via* Belarus from June 2021 to November 30, 2022. In the period 24 February 2022 to 30 November 2022, Lithuania's Immigration Department registered 71,386 Ukrainian refugees. This study investigates the healthcare and social needs of recent asylum seekers who have crossed the Belarusian border and Ukrainian refugees in Lithuania. This is a study protocol for a mixed-methods study which will involve qualitative interviews with asylum seekers who crossed from Belarus in June 2021 and Ukrainian refugees. During a quantitative phase, refugees and asylum seekers will be asked to complete questionnaires. In this study, validated questionnaires will be used, including the Hopkins Symptom Checklist (HSCL-25), the Harvard Trauma Questionnaire (HTQ), and the Short Form 36 (SF-36). Participants will also be asked to self-report sociodemographic information. As a result of the findings of this study, it is possible to provide guidelines for improving access to health care services, including prevention (i.e., vaccination programs) and treatment of chronic and acute illnesses, through primary and secondary healthcare delivery, thereby reducing negative health outcomes. This study may shed light on the social needs of asylum seekers and refugees in Lithuania. In addition, this may provide insight into how they are integrating into the community, such as what their employment and educational prospects are.

## Highlights

- Asylum seekers, refugees, and migrants often do not find their ultimate destinations to be what they had expected. It is widely recognized that a number of the conditions in transit camps, where people often spend months or years, fall below the level of basic humanitarian standards, even within the European Union.
- Access to healthcare varies depending on the country of origin, the reason for immigration, and the length of stay.
- The protocol outlines the research design of a mixed method study investigating the healthcare and social needs of recent asylum seekers who crossed the Belarus borders and Ukrainian refugees in Lithuania.

## Introduction

Asylum seekers, Refugees' and Migrants' (ARM) final destinations are not always what they expect. Many of the conditions in transit camps, where people frequently spend months or years, are below the level of basic humanitarian standards, even in the European Union (EU) (1). A total of 537,355 people applied for asylum in the EU in 2021(2). The significant number of migrants, refugees and asylum seekers presents an additional challenge to the health care system. This is because their health needs coincide with their lack of access to health care (3). Additionally, due to the differences in legal and regulatory frameworks across the EU, asylum seekers, refugees and immigrants have different access to healthcare (4). There are still disparities and inequalities in accessing healthcare, even when legal accessibility is available (5). The access to healthcare services varies for immigrants based on their country of origin, their reason for immigration, and their length of stay (6). Moreover, asylum seekers are at risk of mental illness since they have usually been traumatized by the events and difficulties they have encountered up until the point when their asylum application is granted (7). It has been indicated that social isolation appears to cause mental distress not only because of personality characteristics but also because of several lifestyle elements such as sleep disruption, altered eating patterns, and decreased physical activity (8). It has been suggested that migrants' health vulnerability arises in part from a lack of information about care options (9). In particular, the lack of knowledge about sexually transmitted diseases has been cited (9). It is imperative that refugees and asylum seekers have access to health care providers upon arrival. This will prevent interruptions in medication supply and help reduce the mortality and morbidity associated with cardiovascular, respiratory, and mental illnesses as well as chronic infectious diseases in the host country. Moreover, it is a priority to ensure that the vaccination systems in host countries are fully accessible. For example, Ukraine has historically low vaccination rates among children (10). There are several factors that contribute to perceptions of unmet health needs. These factors include high health care costs, limited awareness of health care systems, culturally insensitive services, stigmas associated with illness, and limited language skills (11). Irregular and undocumented migrants face unique challenges in accessing healthcare services, and since they are unaware of their entitlements, they are likely to receive inadequate care (12). It has been shown that migrants' economic and social circumstances, the harsh conditions in the detention centers, including exposure to cold, lack of space and overcrowding, physical inactivity, poor nutrition, and high levels of stress negatively affect their health (13). These conditions are contributing factors to the majority of health issues in these camps and may be prevented (1).

### Background

ARM across many European countries have found themselves poorly protected and overexposed to the COVID-19 pandemic (14). Governments worldwide struggle to develop and implement policies and legal frameworks that meet the health needs of refugees and migrants (15). A growing body of evidence suggests that migrants in high-income countries are more likely to contract SARS-CoV-2 and to experience a significant number of cases and deaths of COVID-19 (14).

COVID-19 has an impact on migrant populations, including social effects of border closures, longer lockdowns that restrict their movements for work, and long asylum procedures which discourage those who would otherwise want to be reunited with their families (16). Pandemic impacts make them more vulnerable to COVID-19, overcrowded camps and detention centers that may increase exposure to SARS-CoV-2, and lack of consistency in COVID-19 health information strategies for culturally and linguistically diverse communities reduces awareness of prevention measures (16, 17). Fear of arrest is a common reason why undocumented migrants do not seek assistance (17). They experience a lack of access to health care services, mental health problems, and uncertainty about participation in vaccination programs (14). Health authorities are responsible for responding to the COVID-19 pandemic, as well as for implementing vaccine programs. Jordan, for instance, was one of the first countries to include refugees in its public health response during the outbreak, including the national vaccination campaign (18). A holistic approach to the wellbeing of asylum seekers and migrants can be vital to their rehabilitation (19, 20). It is essential that the provision of refuge to Ukrainians be accompanied by measures aimed at ensuring that migrants, regardless of nationality, will be able to obtain quality health care in the future, and training and educating health care professionals, as well as ensuring that displaced persons are aware of their legal rights and where they can obtain health care (10). It has been suggested that further research needs to be conducted on migrants' own perspectives regarding their health and access to healthcare (11).

### Situation in lithuania

The Lithuanian government committed to accepting 1,077 refugees from EU countries as well as third countries in 2015. Six refugees were transferred from Greece and Jordan in 2020. As a result of this agreement, 499 people were resettled in Lithuania from 2015 to 2020 (21). Furthermore, Lithuania granted asylum (refugee status) to 80 individuals in 2020, primarily Russians, Turks, and Tajiks (21).

In 2021, thousands of undocumented migrants were arriving in Latvia, Lithuania, and Poland *via* Belarus. In a short period, a large number of migrants arrived in Lithuania, posing an unprecedented challenge to the government and authorities (22), and 90% of them have requested asylum (23). After crossing the Lithuanian border irregularly, those immigrants are housed at the Foreigners Registration Center (URC), where they are tested for COVID-19 (24). More than 40 nationalities are represented among the group of immigrants, the majority of whom are from Iraq. Apart from families with children, the group also includes unaccompanied minors, elderly individuals, and people with disabilities and serious health issues (23).

The following characteristics are observed among irregular immigrants arrived in Lithuania from June 2021 till November 2022 (22). There were 4,537 undocumented migrants. The majority originate from Middle Eastern countries (Iraq and Syria) and 90% of them applied for asylum. Undocumented migrants from Iraq topped the list of countries of origin for migrants with a total of 2,864 persons, and 190 from Syria. According to gender, there are 1,276 females (28%), and 3,261 males (72%). A foreign national who is a permanent resident in Lithuania and/or who is insured by Lithuania's compulsory health insurance plan (for example, those seeking asylum or refugees) can obtain vaccination services (i.e., COVID-19) in Lithuania, as can all insured citizens of other EU member states (25).

A report by the World Health Organization identified the need for immediate and sustainable solutions to meet the health needs of undocumented migrants entering Lithuania from Belarus. Undocumented migrants arriving from Belarus required medical care, medication, psychosocial support, and information in their native language. Additionally, COVID-19 cases were on the rise throughout the region, so it was crucial to ensure that there was sufficient shelter, hygiene facilities, food, and medicine, as well as access to COVID-19 testing facilities (26).

Between the beginning of the war and November 2022, over 7 million and 8 hundred thousand Ukrainian refugees were registered throughout Europe (27). Furthermore, more than 7 million people are internally displaced within the country during the same period (28). Primarily women, children, and elderly men entered the EU, as the Ukrainian government requires men between 18 and 60 to remain in Ukraine and contribute to the war effort (29). The conflict has forced not only Ukrainians to flee, but students, workers, and refugees from other nationalities (29). There are around **338** thousand third country nationals who have fled Ukraine (30). In addition, not all non-Ukrainians are covered by the Temporary Protection Directive, and those that are, are required to verify their ability to stay in their host country (29). Millions of children are in need of humanitarian assistance as they continue to suffer the war consequences (31).

In a statement released by the International Organization for Migration (IOM), information from partners and humanitarians at border crossings with neighboring countries was verified showing discrimination against several third-country nationals entering neighboring countries to Ukraine (32). The report also outlines incidents of xenophobia based on ethnicity, race, and nationality (32). Lithuania's Immigration Department registered 71,386 Ukrainian refugees between 24 February 2022 and 30 November 2022 (33). Upon arrival in Lithuania, all Ukrainians are directed to a registration center. The five Registration Centers are in Vilnius, Alytus, Marijampole, Klaipeda, and Šiauliai, where shelter, food, clothes, and medical care will be provided and then those in need will be taken to long term accommodation (34).

## Theoretical framework

A social determinant of health (SDH) is defined as the set of conditions under which a person is born, grows, works, lives, and ages (35). These conditions are determined by distributions of money, power, and resources at the global, national, and local levels (35). There is a possibility that SDH is at the core of health inequities (36). This study is based upon the Commission on Social Determinants of Health (CSDH) framework (37). It is hoped that this framework will assist in understanding how political and socioeconomic policies impact the health and social needs of migrants. The Social Determinants of Health (CSDH) framework was developed by the World Health Organization's Commission on Social Determinants of Health to facilitate the analysis and communication of the complex phenomenon of social determinants of health (37).

The CSDH framework distinguishes two types of determinants: structural and intermediary.

There is evidence that structural health determinants are influenced by culture and social values (e.g., religion), public policies (e.g., healthcare) and political systems, all of which influence a range of “structural mechanisms” such as income, education, and occupation (37).

These structural mechanisms, therefore, reproduce and sustain social stratification, determine and reinforce the power and privileges of people, and reinforce their interactions. In many societies, gender discrimination is a fundamental feature. Women and girls are subjected to systematic discrimination when it comes to power, prestige, and resources. Discrimination has the potential to cause immediate and severe health effects (e.g., rape, sexual exploitation, forced marriages, and gender-based domestic violence). Belonging to a marginalized racial or ethnic group has a profound impact on a person's social status, economic prospects, and course throughout life in societies marked by racial discrimination and exclusion. The health outcomes of oppressed racial and ethnic groups tend to be significantly lower than those of more privileged groups or the general population. Inequity in health is determined by the sociopolitical context and structural mechanisms. Furthermore, these determinants are interconnected with another set of determinants, which are intermediary determinants of health (37). The intermediary factors are as follows: (1) Environmental factors: neighborhood characteristics, housing quality, and work environment. (2) Socio-psychological factors: psychosocial stressors and stressful living conditions. (3) Behavioral factors including nutrition, physical activity, smoking, and alcohol consumption. (4) Genetic and aging factors. (5) Factors related to the healthcare system, such as accessibility, and affordability of services. As defined in the framework, intermediary determinants are social factors that affect health and are responsible for unequal exposure and vulnerability to harmful factors, such as access to safe drinking water, poor dietary choices, and unhealthy living conditions (37).

Immigration must be viewed as a health determinant itself in order to achieve substantive improvements in health outcomes (38). Moreover, being an immigrant limits a person's behavior and alters the effects of another social positioning, such as socioeconomic status, since the immigrant's relationship with the state and its institutions, such as health care, is ambiguous (38).

### Aim

The purpose of this study is to investigate the healthcare and social needs of recent asylum seekers crossed the Belarus borders and Ukrainian refugees in Lithuania.

### Objectives

To explore the health and social needs of recent asylum seekers as well as Ukrainian refugees.

To describe the impact of COVID-19 on asylum seekers and refugees.

To assess the quality of life and mental health among asylum seekers and refugees.

## Methods

The research will use a pragmatist research philosophy and a mixed-methods design (using qualitative and quantitative methods to collect data) (39). In this study, the primary aim is to explore the healthcare and social needs of the recent asylum seekers who have crossed the Belarussian border and Ukrainian refugees.

## Data collection

Semi-structured interviews and a quantitative cross-sectional study will be conducted at asylum seeker centers in Lithuania between May and November 2022. The Lithuanian government runs five centers for asylum seekers who have crossed the Belarussian border into Lithuania (40). Three of those centers are under the control of the Ministry of the Interior of the Republic of Lithuania: the centers in Pabradé, Medininkai, and Kybartai. The last two centers are managed by the Ministry of Social Security and Labor: the center in Rukla and the center in Naujamiestis, Vilnius (40). The research team will obtain permission from the management of these centers to conduct the research study, two members of the research team (RA and RU) will visit the centers to invite the asylum seekers to participate in this research study. Study information sheets will be distributed, and the research team will reach out to the asylum seekers to explain any points regarding the research study that they can introduce. The research team will contact all recently arrived migrants (19 years old or older), who entered Lithuania from the Belarussian border in June 2021 or afterward, regardless their nationality, gender, or current asylum status to invite them to participate in the study in those allocated reception centers. The interviews will be conducted by two doctoral students. Since one of those students is a native Arabic speaker, the interviews can be conducted in Arabic, or in English by the two doctoral students (41). The language of the majority of newly arrived asylum seekers migrants in Lithuania from Belarus since June 2021 is Arabic (22). Refugees from Ukraine who arrived on or after 24 February 2022, who are 19 years old or older, are eligible to participate in this study. The research population sampling methods are purposive sampling and a snowball sampling approach according to Bryman and Bell (42). For the individual interviews, individuals under 19 and those not speaking one of three languages Arabic, English, or Russian are excluded. Quantitative phase exclusion criteria include individuals under the age of 19 and those who cannot fill out the questionnaires in one of the available languages.

### Qualitative phase

The information sheet (Supplementary Annex 1) as well as the consent form (Supplementary Annex II) were prepared in English, Arabic, and French. The semi-structured interview guide (Supplementary Annex III) was developed by the research team in English based on previous literature and extensive discussions between the research team members. The guide is translated into Arabic and Russian and then back-translated to ensure accuracy and quality by two independent bilingual speakers (43). The guide represents questions or topics or any other objectives that need to be discussed by the researchers. The interviewer will ask several follow-up questions depending on the responses of the participants to collect maximum data for analysis. A variety of topics will be discussed, such as healthcare access, existing healthcare-related needs, and communication challenges, diet and meals concerns, social activities, social welfare, COVID-19 information available in their native languages, and adherence to health advice, as well as obstacles to integration in Lithuania. Interviews will be audio recorded and transcribed verbatim. In addition, field notes, such as demographics of the participants and key points from each interview, will also be taken during the interviews and referred to later in reflective conversations between the researchers (42). The total number of semi-structured interviews with the asylum seekers will be 16 or more to ensure data saturation for quality responses and conclusions. Similarly, for Ukrainian refugees, a minimum of 16 semi structured interviews will be conducted in the Russian language. It is expected that the interviews or the questionnaire will take ~30–40 min.

### Quantitative phase

#### Measures

Sociodemographic data.

Data on sex, age, country of origin, and additional sociodemographic data, including highest educational level and family situation, will be collected by self-report from the participants.

#### Mental health (anxiety, depression, post-traumatic stress disorder)

This study will use the Hopkins Symptom Checklist (HSCL-25), Harvard Trauma Questionnaire (HTQ), and the Short Form 36 (SF-36) to assess different manifestations of mental health, including anxiety, depression, post-traumatic stress disorder (PTSD), and health-related quality of life. It has been shown that these scales possess sound psychometric properties and are frequently used among refugees and in population-based surveys (44, 45).

There are 10 items pertaining to anxiety and 15 items pertaining to depression in the HSCL-25. The items consist of four alternatives ranging from “not at all” (1) to “very much” (4). The items refer to how specific symptoms have bothered or distressed the individual during the previous week. For each item, the mean score is calculated separately for the anxiety and depression subscales (46, 47). The HTQ is structured similarly to the HSCL-25, in that it uses a similar response format and refers to the same time frame (45). Mean item scores will also be calculated.

### Health related quality of life

The short Form 36-Item Health Survey (SF-36) (48) will be used to evaluate the perspectives of the participants on their health. There are 36 items in the SF-36, which are grouped into eight scales. This survey tool is one of the most widely used to assess quality of life (49, 50). The items are physical activity (PA), physical role (PR), bodily pain (BP), general health (GH), vitality (VIT), social activity (SA), emotional role (ER), and mental health (MH) (51). An additional question is included in the SF-36 to collect information about changes in health status in the past year that are not categorized into any scale. There are two-component scales comprising these eight scales: the Physical Component Scale (PCS) and the Mental Component Scale (MCS).

The SF-36, HSCL-25, and HTQ tool is available in English and Arabic (48). In regard to the quantitative study, the questionnaire will be available in three languages (Arabic, English, and Russian). HTQ and HSCL instructions and guidance provided with the license will be used to calculate the cut-off scores. According to HTQ scores, the cut-off value for PSDT symptomatic and asymptomatic respondents will be 2.5. According to the HSCL scores, the cut-off value is 1.75.

Blackmore et al. found that the prevalence of posttraumatic stress disorder (PTSD) among refugees was 31.4% (95% CI 24.43–38.5), depression was 31.5% (95% CI 22.64–40.38), anxiety disorders were 11% (95% CI 6.75–15.43), and psychosis was 1.51% (95% CI 0.63–2.40) (52). Using 31.5% as the prevalence rate for depression and PTSD, the *p* and *q* values of the formula are 0.315 and 0.685. Therefore, 332 samples are required.

### Data analysis

#### Qualitative data

ATLAS-ti qualitative software will be used (53) to transcribe the interview data. Interviews will all be transcribed verbatim. Interviews will be thematically analyzed (54). The first step involves a member of the research team reading and rereading the transcripts to gain immersion in the data, highlighting key quotes, and establishing codes based on the participants' verbatim statements. The codes will be grouped into sub-themes and themes in accordance with common threads evident throughout the data. A second researcher will review an electronic audit trail of this process to ensure that the interpretation of data and the organization of codes are consistent. The two researchers will discuss disagreements or contested themes/subthemes/theoretical domains until agreement is reached. The process of analyzing the data will be iterative, with the researchers constantly referring back to the raw data in order to validate and affirm emerging themes and ideas. The anonymity of participants will be protected by using pseudonyms throughout the study.

#### Quantitative data

The Shapiro–Wilk test will be used to determine whether the distribution of the variables is normal. To compare the means with the normal distribution of variables, a *T*-test will be used. A Mann–Whitney *U*-test will be applied for variables that do not comply with the assumption of normality. Frequencies between the groups will be compared using the χ^2^ criterion. Significance level α = 0.05 is chosen for statistical analysis. Measures of central tendency are presented as follows: mean ± standard deviation, for variables with normal distribution, or median (first quartile–third quartile) for variables with other distribution.

### Trustworthiness

A pilot study will be conducted to determine the validity of the qualitative interview guidelines and the quantitative questionnaires. This study will adhere to the scientific principles of qualitative descriptive phenomenology to ensure validity and reliability (55, 56). Hence, to avoid bias, researchers need to be conscious of their preexisting knowledge about the world, of their relationships with those around them, and also of the risk of giving voice to themselves rather than the participants (55, 56). To minimize bias, all researchers engage in critical discussions of their preconceived notions and theoretical knowledge throughout the research process (55, 56). No researchers will be familiar with the participants covered by the research. Detailed explanations of how sub-themes and themes are abstracted and interpreted will allow readers to determine whether the study is valid and trustworthy, and the findings will be supported by excerpts from all original interviews (55, 56). Detailed explanations of how sub-themes and themes are abstracted and interpreted will allow readers to determine whether the study is valid and trustworthy, and the findings will be supported by excerpts from all original interviews (55, 56). An analysis of the internal consistency or reliability of the completed questionnaires will be made by using the Cronbach's Alpha test.

## Discussion

The purpose of this study is to gain a broader understanding of the health and social needs of newly arrived asylum seekers and Ukrainian refugees in Lithuania. The research team anticipates that Lithuania will experience another large influx of immigrants from Europe or non-European countries. Therefore, the study findings could provide guidance as to how to improve access to health care services, including prevention (such as vaccination programs) and treatment of either acute or chronic diseases through both primary and secondary care, which may reduce the negative health effects of the immigration process. In this study, the research may be able to shed light on the social needs of immigrants in Lithuania and their integration into the Lithuanian community, such as job prospects, learning the Lithuanian language, or entering schools for children and academic institutions for immigrants. It may be possible in the future to carry out research that reconsiders the needs of immigrants, to work more closely with immigrant communities, and to disseminate programs that are tailored in keeping with the actual needs and realities of immigrant communities.

One of the challenges facing this study is that it involves several migrant groups. As an example, the asylum seekers who crossed the Belarussian border into Lithuania represent more than 40 different countries, along with the various languages they speak. The research team decided to conduct the interviews only in Arabic and English. The questionnaires will be available in Arabic and in English. In regard to refugees from Ukraine, a language barrier also exists since none of the members of the research team speak Ukrainian. It has been decided to conduct interviews and questions in Russian, which is widely spoken by Ukrainians, however, it may be sensitive for the research team to request that Ukrainians complete interviews and questionnaires in Russian. Funding is certainly of vital importance to the success of such an endeavor. Despite this, no funding opportunities could be identified in such a short period of time. The project will proceed with the limited resources of the research team.

## Data availability statement

The original contributions presented in the study are included in the article/Supplementary material, further inquiries can be directed to the corresponding author.

## Author contributions

All authors listed have made a substantial, direct, and intellectual contribution to the work and approved it for publication.
