# Supplementary material for: Health and social needs of asylum seekers and Ukrainian refugees in Lithuania: A mixed-method protocol
Source: Front Public Health. 2023 Jan 10;10:1025446. doi: 10.3389/fpubh.2022.1025446 (PMC9875536; doi:10.3389/fpubh.2022.1025446)
Supplement: Supplementary file 1 [file Data_Sheet_1.docx]

**Annex1**

**Information sheet for participants: Individual interviews**

Project title: Healthcare services and social needs of recent asylum seekers and Ukrainian refugees entering Lithuania

**Purpose of this document**

The xxx would like to invite you to participate in a research study. This information sheet provides you with the information about the research performed – its reasoning, advantages and disadvantages of participation as well as other important information. Please read the following information carefully and discuss it with your family members, friends, or colleagues, if desired. If you have any questions, feel free to contact us. If you decide to participate in this research, you will be asked to join an individual interview. Withdrawal is possible at any time without denoting your reasons.

**What are the objectives of the study?**

The study is divided into four substudies, as follows:

Asylum seekers and refugees in Lithuania are being interviewed individually to identify their social and healthcare needs. We will also be conducting focus group discussions with representatives of governmental and non-governmental organisations in Lithuania to better understand the challenges and facilitators Lithuania encounters when providing health services to asylum seekers and migrants who entered the country through Belarus and refugees from Ukraine. In addition, one of our objectives is to collect data about the self-reported health status of migrants seeking asylum in Lithuania, potential health risk factors, and their utilization of health care services. In light of the increasing number of refugees and asylum seekers in Lithuania, we are planning to assess the level of cultural competence of nurses working in the nursing field at present.

**Do I have to participate?**

Participation is completely voluntary. It is entirely up to you whether or not you wish to participate. Involvement in any community group or service will not be affected by your decision to participate.

**If I participate, what will happen to me?**

 If you choose to participate, you will be invited to attend a meeting with a member of our research team and an interpreter if necessary. The interviewer will use an interactive format to discuss the topic “What are the healthcare and social needs of migrants and asylum seekers?”

You will not have to discuss anything that makes you uncomfortable. The meeting will last approximately 40 to 60 minutes. There will be an audio recording of the meeting, and the interviewer may take some written notes.

**Is my participation in this study confidential?**

This study has been approved by the xxxxx).  Participation in this study, as well as everything you tell us, will be kept strictly confidential. Your name and contact information will be removed from any information we maintain and will only be identifiable by an ID number. A member of the research team will transcribe the contents of the recording following the interview. The xxxx will maintain all electronic information in password-protected files. Access to this information will be restricted to the research team only.

**What are the advantages and disadvantages of participating?**

In this study, you will be able to discuss your experiences and needs regarding health and social services in Lithuania. In the meeting, you will be able to discuss what you think is relevant and what you think should be improved. This will be valuable for determining how to meet the needs of every individual in Lithuania

It is possible that some individuals will find some of the topics discussed to be difficult or upsetting. You may withdraw from the meeting at any time, and you are not required to discuss anything that you feel uncomfortable discussing.  According to academic research standards, there is no financial compensation for participants in the study.

**What will happen to the research study's results?**

The results of this study will be published in academic journals and the findings will be communicated to relevant governmental and non-governmental organizations as well as to relevant conferences and seminars at both the national and international levels. You cannot be identified from any of these publications, as mentioned earlier. Results will be made available to the participants.

**Contact persons to ask questions**

Regarding your rights as a study participant or other questions, you can contact the following researchers:
