# Supplementary material for: Health and social needs of asylum seekers and Ukrainian refugees in Lithuania: A mixed-method protocol
Source: Front Public Health. 2023 Jan 10;10:1025446. doi: 10.3389/fpubh.2022.1025446 (PMC9875536; doi:10.3389/fpubh.2022.1025446)
Supplement: Supplementary file 2 [file Data_Sheet_2.docx]

**Annex** II

**Informed consent**

I agree to participate in the study entitled "Healthcare services and social needs of recent asylum seekers entering Lithuania from Belarus and Ukrainian refugees." I will take part in an interview as part of my participation. My participation in the study is completely voluntary, and I have been informed that I may withdraw at any time during the study.

This information has been read by me. I have had the opportunity to ask questions about it. My questions have been satisfactorily answered. I consent voluntarily to participate in the study.

Name of Participant ________________________________

Signature of Participant ________________________________

Date ________________________________

Day/month/year
