# Supplementary material for: Health and social needs of asylum seekers and Ukrainian refugees in Lithuania: A mixed-method protocol
Source: Front Public Health. 2023 Jan 10;10:1025446. doi: 10.3389/fpubh.2022.1025446 (PMC9875536; doi:10.3389/fpubh.2022.1025446)
Supplement: Supplementary file 3 [file Data_Sheet_3.docx]

Annex III

**Interview guide**

The participant should be thanked for his or her interest in contributing to the research study.

Presentation of the researcher/interviewer and the interpreter, if present

A presentation of the study's objective, its purpose, and the intended use of the findings.

Describe how the interview will be conducted.

The participant should read and sign the informed consent form.

**Information regarding the background:**

Country of origin, age, and educational level

**Begin with an open question:**

In terms of health and social welfare, what expectations did you have before you went there? Did these expectations match your current experiences?

Positively, what did you find? What did you find negative?

How did you experience the treatment of any health problems you had?

How much information did you receive about health care and how to obtain care? How was the information regarding your right to health care and social welfare provided to you?

What was your experience with the information about COVID-19? Was anything particularly noteworthy? Was anything missing?

What improvements can you suggest?

**Based on the direction the interview takes, the following questions may lead back to the topic**

**Answers from participants will be followed by follow-up questions to encourage them to share more and to clarify their views.**

What are the cultural differences between Lithuania and your home country? What effect has this had on you?

What is the importance of learning Lithuanian to you? If you have begun to learn Lithuanian, how did you do it? How have you found the learning process?

 How do you feel about your living space? Are you satisfied with it?

When you have spare time, with whom do you spend it? What kind of activities do you engage in?

Is there anyone you can ask for support or assistance if you need it in your immediate surroundings?

Would you like to obtain a job/education? If so, why? What kind of job/education would you like? What options do you envision for your future employment? Could you describe the benefits of obtaining a job/education?

According to you, what are the biggest obstacles to integration in Lithuania?

Would you like to add anything to the discussion that has not been addressed so far?

**When the participant has nothing further to say, the interview is ended with the researcher providing a summary to ensure the researcher has understood the participant correctly.**

**Ask the participant to clarify any misunderstandings or add more information.**

**Afterward, the researcher thanks the participant, and the interview concludes.**
